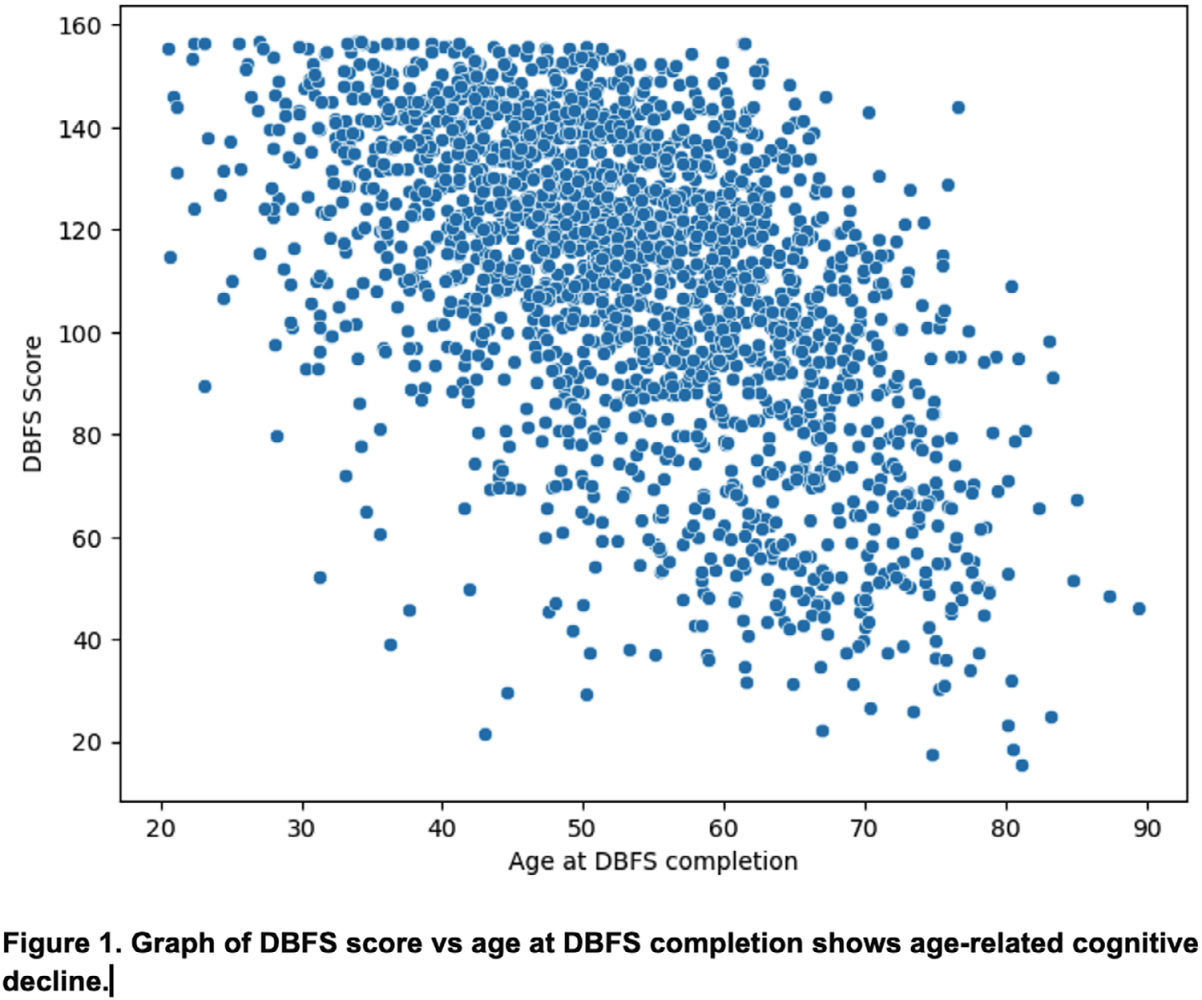# Modifiable Risk Factors for Mild Cognitive Impairment in Singapore: Real‐World Findings from Community‐Dwelling Singaporeans using the Digital Brain Function Screen (DBFS)

**DOI:** 10.1002/alz70857_101882

**Published:** 2025-12-25

**Authors:** Nav Vij, Jermyn Z See, See Ann Soo, Prem Pillay

**Affiliations:** ^1^ Neurowyzr Pte Ltd, Singapore, Singapore, Singapore; ^2^ Singapore Brain and Spine Specialist Singapore Brain‐Spine‐Nerves Center, Singapore, Singapore, Singapore

## Abstract

**Background:**

Mild cognitive impairment (MCI) represents a transitional stage between normal cognitive aging and dementia, characterized by a discernible decline in cognitive function that does not substantially impede daily activities. It serves as a critical intervention point where early detection and lifestyle modifications can delay progression. Globally, dementia‐related costs may exceed US$2 trillion by 2030. In Singapore, around 10% of seniors have dementia, with costs projected to surpass S$1 billion by 2030. Neurowyzr's Digital Brain Function Screen (DBFS) identifies early MCI signs alongside modifiable lifestyle risk factors, offering a pathway for intervention.

**Method:**

DBFS is a medical grade digital cognitive screening tool that assesses cognitive function through tasks spanning four domains: attention, executive function, immediate memory, and working memory. Additionally, a lifestyle questionnaire captures data on physical activity, sleep, nutrition, alcohol intake, smoking, other medical conditions and medications, and brain stimulation. Over 2,000 individuals from routine health screenings in Singapore completed the DBFS, providing real‐world data. Statistical modeling identified key factors linked to MCI, distinguishing cognitively healthy individuals and those at high risk of developing MCI or dementia.

**Result:**

DBFS scores declined with increasing age, indicating age‐related cognitive decline (Figure 1). Diabetes was strongly linked to poorer cognitive performance, while smoking and daily sugar consumption (≥1 teaspoon) were significant MCI risk factors. In contrast, regular physical activity—especially cardiovascular and resistance training thrice weekly—was neuroprotective. Higher education (bachelor's degree or higher) provided added protection, particularly in older adults. Additionally, engagement in cognitively stimulating activities at work or leisure further reduced the likelihood of cognitive decline.

**Conclusion:**

These real‐world findings underscore the multifactorial nature of MCI in Singapore and highlight critical modifiable risk factors. The DBFS shows promise as a scalable tool for early detection and targeted prevention strategies—managing diabetes, reducing smoking, limiting sugar intake, and promoting regular exercise, educational attainment, and cognitive stimulation. Early identification and intervention may mitigate MCI risk and help reduce the overall economic burden of dementia.